# On the *Leucanthemopsis alpina* (L.) Heywood growing in the Illyrian region

**DOI:** 10.3897/phytokeys.161.53384

**Published:** 2020-09-15

**Authors:** Salvatore Tomasello, Kamil Konowalik

**Affiliations:** 1 Department of Systematics, Biodiversity and Evolution of Plants (with Herbarium), Albrecht-von-Haller Institute for Plant Sciences, Untere Karspüle 2, 37073, Georg-August University, Göttingen, Germany Georg-August University Göttingen Germany; 2 Institute of Biology, Wroclaw University of Environmental and Life Sciences, Kożuchowska 5b, Wroclaw, Poland Wroclaw University of Environmental and Life Sciences Wroclaw Poland

**Keywords:** alpine plants, Compositae, endemism, flora of Bosnia and Hercegovina, glacial refugia, Vranica Mts

## Abstract

*Leucanthemopsis alpina* (L.) Heywood (Asteraceae, Anthemideae) is a small, caespitose plant growing in high alpine environments in all the main southern European mountain ranges. However, the species status in the Balkan Peninsula (and especially in the Dinaric Alps) is not very well known. Surrounding this area, different *L. alpina* subspecies are found in the Eastern Alps and in the Carpathians. These subspecies differ from one another, both morphologically and in chromosome number. The present study aims to better characterise the populations of *L. alpina* in the Illyrian and Balkan regions by undertaking a comprehensive survey of herbarium collections for the species in this area, by applying flow cytometry for ploidy determination and by sequencing of two chloroplast markers. Results from our investigation suggest that the only population of the species in the Dinaric Alps is found in the Vranica Mts (Bosnia and Herzegovina). This population consists of diploid plants (unlike tetraploid populations from the Eastern Alps) that are slightly distinct genetically from those of the subspecies growing in the Eastern Alps and the Tatra Mts. Both the ploidy and their genetic distinction indicate that Vranica Mts most probably served as a refugium for the species during the Pleistocene glaciations. Considering its isolated geographical range and its genetic distinction, the population of *L. alpina* growing in the Vranica Mts should be considered as a separate subspecies.

## Introduction

*Leucanthemopsis alpina* (L.) Heywood (Asteraceae, Anthemideae) is a small, caespitose, scapose perennial herb that grows in high alpine environments at elevations of (1800–) 2000–3600 m (Tomasello and Oberprieler 2017). It is a silicicolous species growing in shallow clastic alpine soils, screes and ridges. It is widely distributed across all the main southern European mountain ranges, from the Pyrenees to the Carpathians. It does not extend extensively into the main Mediterranean peninsulas. Seven subspecific taxa are known and three ploidy levels have been recorded across its distribution range (i.e. diploids, tetraploids, and hexaploids; Tomasello and Oberprieler 2017). It is completely missing from the Iberian Peninsula; a hexaploid *Leucanthemopsis* population from the Sierra de Úrbion (i.e. L. alpina subsp. cuneata (Pau) Heywood = *L. cuneata* (Pau) Holub) is not considered to be part of the species; this has been amply demonstrated by earlier studies (Holub 1977; Tomasello 2014; Pedrol 2019). In the Italian Peninsula, the species is found as a single population in Mt. Prado (Tuscan-Emilian Apennines). In the Balkan Peninsula, the species is present in the Southern Carpathians and the Dinaric Alps, although until this study was completed, the species’ status was unclear for the latter area. Beck et al. (1983) reported the presence of *L. alpina* in the Vranica Mts and Mt. Maglić (Bosnia and Herzegovina). However, Mt. Maglić is a calcareous mountain and, therefore, the bedrock is unsuitable for the species. In the Red List of the flora of Bosnia and Herzegovina (EU Greenway Sarajevo 2013), the status of the species is given as uncertain due to the lack of information. Recently, the species was collected on Mt. Jahorina (Bosnia and Herzegovina) and included is a study aiming at investigating the genome size of several taxa from the Balkan Peninsula (Siljak-Yakovlev et al. 2010). In that study, a genome size of 2C = 25.13 pg (SD: 0.32) was reported for the species, which would be indicative of hexaploid plants in *L. alpina* (Tomasello & Oberprieler, 2017). The only confirmed hexaploid populations of *L. alpina* are known for a restricted area of the Central Pyrenees (La Maladetta, Aragon, Spain). Adjoining the Illyrian region, *L. alpina* is present in the Eastern Alps, in the Tatra Mountains and in the Southern Carpathians. Populations from the Eastern Alps are tetraploids and are represented by the subspecies cuneifolia (Murr) Tomasello & Oberpr. In the Tatra Mountains, *L. alpina* plants are diploid and are represented by the subspecies tatrae (Vierh.) Holub. Populations from the Southern Carpathians are diploid and their subspecific status is uncertain.

With the present study, we aim at a better characterisation of *L. alpina* in the Illyrian region, by undertaking a comprehensive survey of the herbarium collections made in this area. We applied flow cytometry to freshly-collected material to estimate ploidy level and sequenced two chloroplast intergenic spacers for a more precise genetic characterisation of the Illyrian populations in comparison with other infraspecific taxa of the species.

## Materials and methods

### Plant material

**Figure 1.**
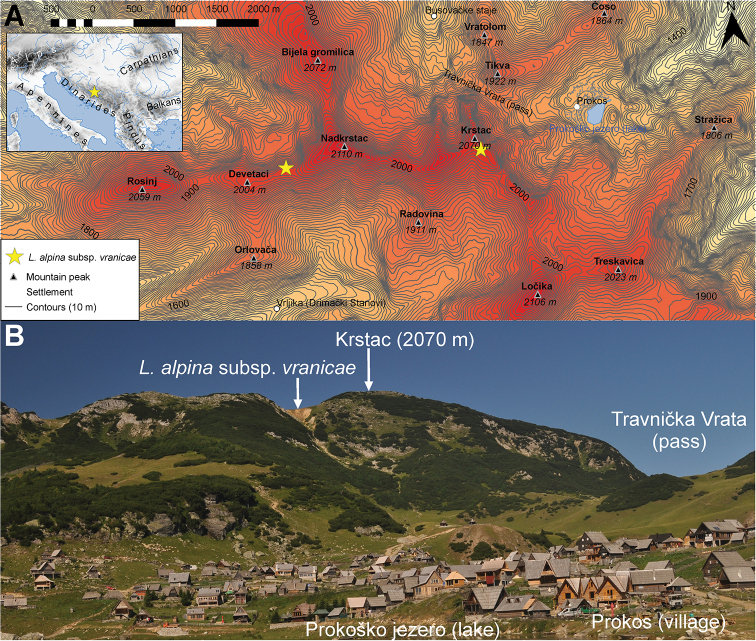
**A** Map showing two locations of Leucanthemopsis alpina subsp. vranicae found in 2018. The map encompasses the central ridge of the Vranica Mts with main peaks and distinguishable topographic features. The contours are spaced at 10 m with every 100 m bolder. The elevation data comes from the ALOS DEM dataset (Tadono et al. 2014). Map is projected in ETRS89 (EPSG: 3035). **B** View from Lake Prokoško towards eastern slopes of Krstac (2070 m). From this point, Nadkrstac (2210 m) the highest peak of the Vranica Mts is behind Krstac. Arrow points to the richest population of L. alpina subsp. vranicae found during the excursion in 2018 on the edge of the bluff close to the Krstac peak. Solitary plants were also found along the path from Krstac to Devetaci. It is likely that it also grows on the inaccessible northern slopes of those mountains.

We consulted the most important herbaria housing conspicuous specimens from the Balkan region (B, W, WU, M, GZU, BRNU, BEOU and SARA). We checked all specimens of *L. alpina* coming from the region, revised them in case of misidentification and databased label information for georeferencing of collection localities. We also used the JACQ virtual herbaria (https://herbarium.univie.ac.at/database/) and GBIF (https://www.gbif.org/) to search for additional herbarium vouchers and distribution data for the species in the region. An additional population was sampled in Nadkrstac Mt (Vranica Mts, Bosnia and Herzegovina; Fig. 1, Suppl. material 1, Table S1). For this population, leaf material from five individuals was collected and dried in silica-gel for subsequent flow cytometric analyses and DNA extraction.

### Flow cytometry

Ploidy was determined by flow cytometry for the five silica-gel-dried samples collected in the Vranica Mts. Petunia × hybrida E. Vilm. ‘P × Pc6’ (2C = 2.85 pg; Marie and Brown 1993) was used as the internal standard. For each measurement, circa 0.5–1.0 cm^2^ leaf tissue of the standard and about twice this amount of leaf tissue from the *Leucanthemopsis* samples were utilised. Nuclei were isolated in Otto I buffer (Otto 1992; Doležel and Göhde 1995) and subsequently stained with 4’,6-diamidino-2-phenylindole (DAPI) in Otto II buffer (Otto 1992; Doležel and Göhde 1995). Samples were then measured on a PARTEC CyFlow Space (Partec GmbH, Münster, Germany) and the results were analysed using the software FloMax (Partec GmbH, Münster, Germany). For each sample, > 8500 nuclei were counted. The relative fluorescence intensity of DAPI-stained nuclei was registered and the ratio between the relative fluorescence of the sample and the standard was used as DNA content estimate to infer ploidy. Two samples were re-measured and used as replicates to test the reliability of the measurements.

### DNA Extraction, amplification, and sequencing

Genomic DNA was extracted from ~1.5 cm^2^ leaf material of silica-dried samples using the Qiagen DNeasy Plant Mini Kit (Qiagen, Hilden, Germany). The plastid intergenic spacer regions *psbA-trnH* and *trnC-petN* were used for building a haplotype network, based on the five accessions from the Vranica Mts and 16 additional populations (three samples per population) from different subspecies and areas of the species distribution range. Sequences for the latter samples have already been used in Tomasello and Oberprieler (2017) and are publicly available in GenBank (https://www.ncbi.nlm.nih.gov/genbank/; see Suppl. material 1, Table S1).

Polymerase chain reaction (PCR) was performed using the primers psbAf and trnHr (Sang et al. 1997) for *psbA-trnH* and trnC (Demesure et al. 1995) and petN1r (Lee and Wen 2004) for *trnC-petN*. The former marker was amplified using the Biotaq polymerase (Bioline, London, UK), whereas the DreamTaq polymerase (TermoFisher Scientific, Waltham, USA) was used for the marker region *trnC-petN*. Reaction mixes were prepared according to the respective manufacturer’s recommendations. For both marker regions, we used the following temperature profile: 95 °C for 3’; 30 cycles of 30” at 95 °C, 1’ at 60 °C and 1’ at 72 °C; and a final elongation step at 72 °C for 5’. PCR products were purified with the HighPrep magnetic beads (MagBio, Gaithersburg, USA). We used 36 µl of HighPrep for 20 µl of PCR product. Finally, samples were sent to Microsynth-Seqlab (Göttingen, Germany) for sequencing. Electropherograms were analysed in FinchTV 1.3.1 (Geospiza Inc., Seattle, USA). The newly-obtained sequences were aligned by hand to those of Tomasello and Oberprieler (2017) in AliView 1.18 (Larsson 2014). A table with information for all the populations from Tomasello and Oberprieler (2017) included in the haplotype network analysis along with GenBank accession numbers is given in Suppl. material 1, Table S1. Indels were coded as binary characters using the simple gap coding method of Simmons and Ochoterena (2000) as implemented in GapCoder (Young and Healy 2003). The two alignments were finally concatenated and used to reconstruct the plastid haplotype network with TCS Version 1.13 (Clement et al. 2000), following the statistical parsimony algorithm of Templeton et al. (1992). We used the web-based programme tcsBU (Múrias dos Santos et al. 2015) for editing and producing a graphical representation of TCS haplotype network.

## Results and discussion

A complete list of the *Leucanthemopsis alpina* vouchers found in the examined herbaria is given in Table 1. From all specimens examined, we could confirm five collections, all from the Vranica Mts (Bosnia and Herzegovina). Two collections from the Herbarium of the National Museum of Bosnia and Herzegovina (SARA-42314, SARA-42312) were misidentified and referable to *Anthemis cretica* L. agg. In the JACQ virtual herbaria, three specimens were reported from WU, BRNU and GZU. A collection made in the Vranica Mts from WU was confirmed as *L. alpina*. A collection from BRNU was a misidentified specimen of A. cretica subsp. carpatica (Willd.) Grierson. We were not able to see GZU-285293, a specimen collected in Durmitor Mt., which is a calcareous mountain further to the south and, therefore, an unlikely locality for *L. alpina*; this may be a misidentified specimen. Across its distribution range, plants belonging to the *A. cretica* agg. and/or (more rarely) alpine *Achillea* species (e.g. *Achillea oxyloba* (DC.) Sch.Bip. and Achillea erba-rotta subsp. moschata (Wulfen) Vacc.) are often mistaken for *L. alpina*. However, with a closer look, it is relatively easy to distinguish these species from *L. alpina*. In contrast to *L. alpina*, *Anthemis cretica* s.l. has bipinnatisect leaves (at least some leaves) and paleaceous receptacles. *Achillea* species have an overall different appearance to *L. alpina*, capitula are rarely solitary and (as for *Anthemis*) the receptacles are paleaceous.

**Table 1.** A complete list of the *Leucanthemopsis alpina* vouchers from the Illyrian region found in the examined herbaria, in the GBIF and in JACQ Virtual Herbaria. Information is provided on herbaria, specimen herbarium-codes, collection locality, collector (when given/readable), date of collection, country and, as necessary, correct identification. Country codes are following the ISO 3166-1 alpha-2 code. Accordingly, BA is for Bosnia-Herzegovina; ME is for Montenegro; RS is for Serbia.

Herbarium	Code	Locality	Collector	Date	Country	Correct identific.
WU	WU 0043817	Vranica	Brandis, E. s.n.	26.07.1888	BA	
SARA	SARA 42315	Maglić	Hamelka s.n.	July 1890	BA	*Anthemis cretica*
SARA	SARA 42314	Maglić		July 1889	BA	*Anthemis cretica*
SARA	SARA 42312	Vranica	Brandis, E. s.n.	26.07.1888	BA	
SARA	SARA 42311	Krstac (Vranica)		August 1919	BA	
SARA	SARA 43310	Krstac (Vranica)	Reiser, O. s.n.	13.08.1901	BA	
GZU		Vranica	Brandis, E. s.n.	09.07.1901	BA	
GZU	GZU 285293	Durmitor Mts	Lanz Josefine & Rauch Cornelia s.n.	17.06.2011	ME	?
BRNU	BRNU 653502	Midjor Mt.	Grulich et socii	09.06.2015	RS	*Anthemis cretica*

Almost all specimens from the Vranica Mts were collected by Erich Brandis. Erich Maria Heinrich Joseph Franz von Sales “Graf zu Brandis” was a Jesuit priest, who served as a professor for almost 40 years in the Archdiocesan Seminary and Secondary School in Travnik (Szeląg 2015). He carried out floristic, faunistic and geomorphological research in Bosnia-Herzegovina and contributed to the collections (especially plants and Coleoptera) of the National Museum in Sarajevo (Hörnmann 1893). Brandis (1891) in “Botanische Beiträge zur Flora von Travnik in Bosnien” cites Vranica as the only locality for *Chrysanthemum alpinum* L. (= *Leucanthemopsis alpina*) in Bosnia-Herzegovina.

Results from flow-cytometric measurements are reported in Table 2. The difference in sample/standard ratio between independent replicates was on average 0.035. For all measurements, the average coefficient of variation (CV) was 4.77% (±0.7) for the internal standard (*Petunia hybrida*) and 5.31% (±0.7) for the *Leucanthemopsis* from the Vranica Mts. All measured samples show sample/standard ratio ranging from 3.46 and 3.83 (mean = 3.69; SD = 0.02). According to these measurements, all samples from Vranica Mts are diploid (Table 2).

**Table 2.** Results from the flow-cytometric measurements, including fluorescence peak for the internal standard *Petunia hybrida*, their coefficient of variation (CV), peaks from *L. alpina* samples from the Vranica Mts (LPS212) and their CV, sample/standard rations and inferred ploidy. ^†^Average value of sample/standard ratios for diploids, tetraploids and hexaploids of *L. alpina* along the whole species distribution range (Tomasello 2014).

Sample	*P. hybrida* peak	CV	*L. alpina* peak	CV	Ratio	Ploidy
*L. alpina* 2x ^†^					3.34	**2**×
*L. alpina* 4x ^†^					5.93	**4**×
*L. alpina* 6x ^†^					7.86	**6**×
LPS212-1	5.42	3.95	20.12	5.22	3.71	**2**×
LPS212-2	5.45	4.97	20.64	3.61	3.79	**2**×
LPS212-3	5.42	4.08	20.74	4.38	3.83	**2**×
LPS212-4	5.43	4.83	19.83	6.02	3.65	**2**×
LPS212-5	4.41	6.04	15.27	7.3	3.46	**2**×

**Figure 2.**
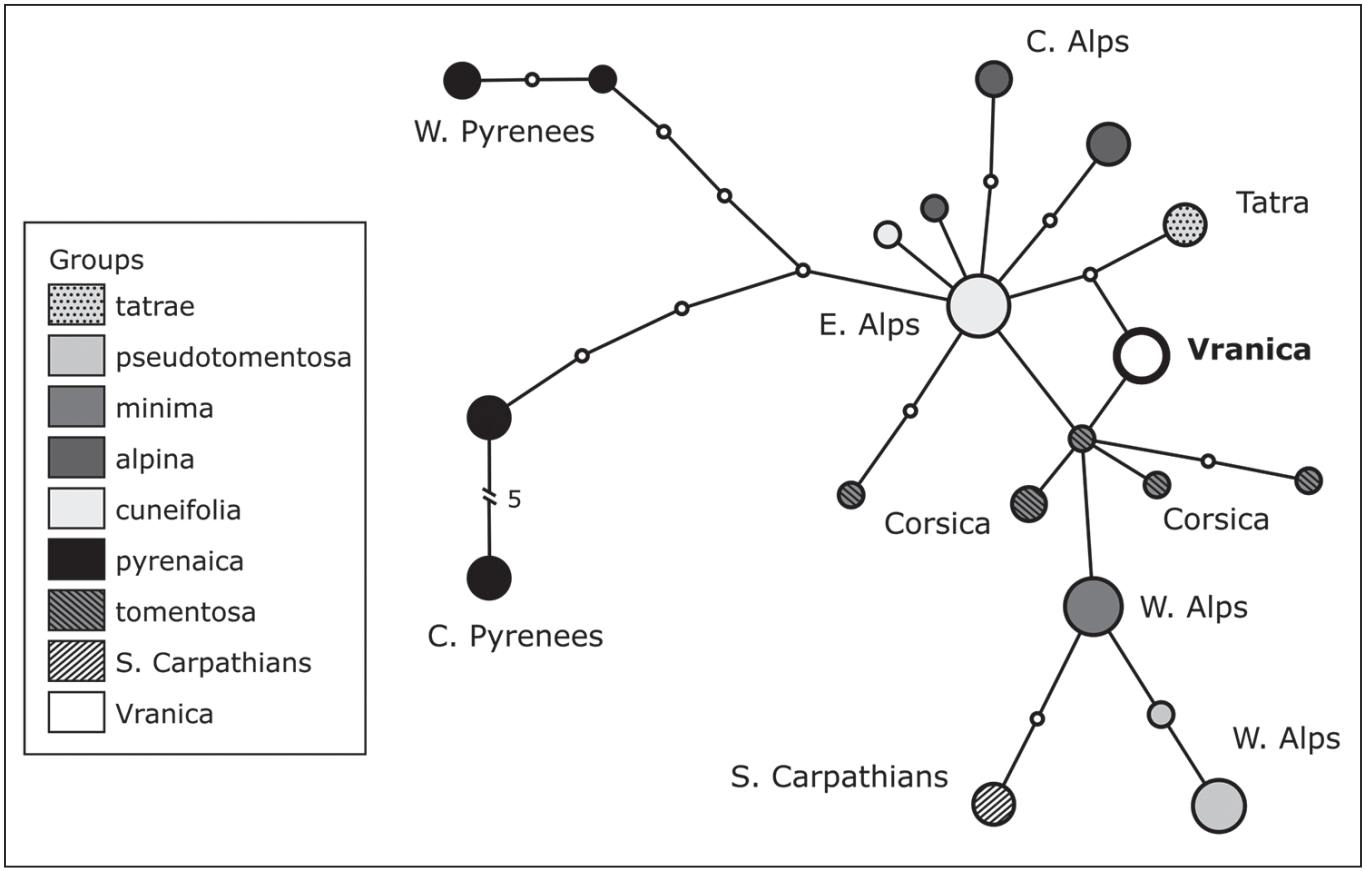
TCS haplotype network obtained from sequencing the plastid intergenic spacer regions *psbA-trnH* and *trnC-petN* for 52 individuals of *L. alpina* from 17 different populations. Circle dimensions are proportional to the haplotype occurrence. Small, white circles represent non-detected intermediate haplotypes. Colours refer to different subspecies and or geographic areas (for populations from the Vranica Mts and the Southern Carpathians). Circle with thick outlines refer to the haplotype registered in the *L. alpina* samples from the Vranica Mts.

The haplotype network is shown in Figure 2. The final concatenated alignment was 1130 bp long (plus nine gap-coding characters) and consisted of 52 sequences from 17 populations. The statistical parsimony network consists of 20 different haplotypes. All samples from the Vranica Mts have the same haplotype, which is found to be equidistant from those found in the Eastern Alps and in the Tatra Mts and also connected to a (presumably older) haplotype found in samples from L. alpina subsp. tomentosa (Loisel.) Heywood (Corsica).

In conclusion, *Leucanthemopsis alpina* is represented in the Dinaric Alps by a single population in the Vranica range. This population is composed of diploid individuals that are genetically differentiated from the closest populations of the species in the Eastern Alps (L. alpina subsp. cuneifolia) and in the Tatra Mts (L. alpina subsp. tatrae). As this population is diploid and has a distinctive haplotype, it is most probable that the Vranica Mts represented a refugial area for the species during the Pleistocene glaciations and, at the present time, the mountains might serve as a warm-stage microrefugium (Gentili et al. 2015).

The above conclusion is congruent with previous research indicating that other refugial populations of Alpine and Carpathian taxa are found within this mountain range (e.g. *Leucanthemum rotundifolium* (Willd.) DC., *Hieracium nigrescens* Willd. (Asteraceae) and *Carex curvula* All. (Cyperaceae); Horvat and Pawłowski 1938; Redžić 2007; Szeląg 2012). Thus, the Vranica Mts represents an important hub for migration between the Alps, the Carpathians and the other ranges on the Balkan Peninsula. Within this massif, there are several endemic taxa (e.g. *Alchemilla vranicensis* Pawł. (Rosaceae), *Edraianthus niveus* Beck (Campanulaceae) and *Viola vranicensis* Beck (Violaceae)), 37 arctic-alpine species (Stevanović et al. 2009) and a high number of plant communities (Redžić 2007) which makes it one of the most important centres of richness and diversity of mountainous vegetation in the Balkan Peninsula (Stevanović et al. 2009). This evidence suggests that it is an important biodiversity hotspot that should be protected against anthropogenic pressure. As such, it is planned as an area of nature protection (Ðug and Drešković 2012). However, even though it represents a stable environment as indicated by a long span of repeated collections, due to its small area, it may be threatened by stochastic events and anthropogenic environmental changes.

**Figure 3.**
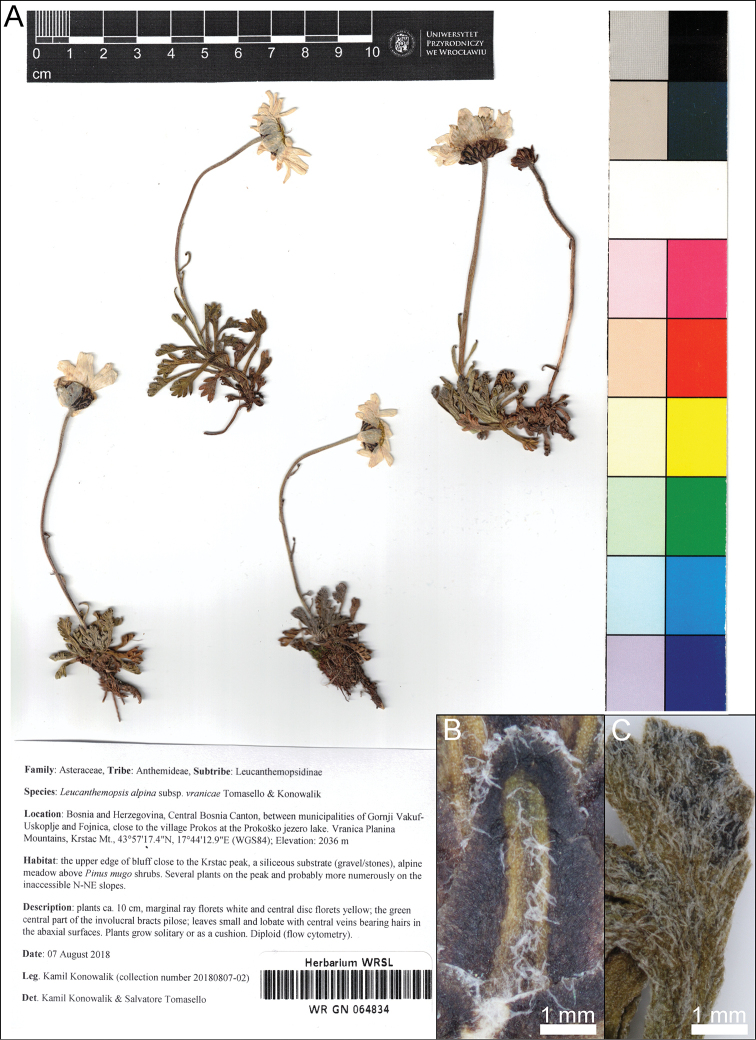
**A** Herbarium voucher (WRSL: WR GN 064834) of the type material from the Leucanthemopsis alpina subsp. vranicae, collected in 2018 by K. Konowalik in the Vranica Mts. **B** Detailed pictures of the involucral bract **C** abaxial face of a basal leaf.

From a morphological point of view and according to the herbarium vouchers observed (see especially leaves incision and leaflet length/rachis width; Figure 3), the population from Vranica Mts is most similar to L. alpina subsp. cuneifolia rather than to the plants growing in the Tatra Mts (L. alpina subsp. tatrae). However, L. alpina subsp. cuneifolia is usually tetraploid (only a few diploid populations are known for the subspecies in the Dolomites; Tomasello and Oberprieler 2017), whereas populations from the Tatra Mts have the same ploidy as *L. alpina* plants in the Vranica Mts. Interestingly, the voucher housed in WU (WU-0043817) was determined as “forma cuneifolium” by Vierhapper, who described different forms in the species (Vierhapper 1914). However, the label identification was then corrected in pencil to “forma tatrae”, although we do not know if this was done by Vierhapper. Hayek (1931) ascribed the *L. alpina* growing in the high mountain pastures of Bosnia and Herzegovina to the variety “*cuneifolia*” (= L. alpina subsp. cuneifolia). A more extensive morphometric study including populations from the Eastern Alps, Tatras and the whole Balkan Peninsula (only one population from the Southern Carpathians was included in Tomasello and Oberprieler 2017) would be needed to better characterise the position of the Vranica population amongst the closest infraspecific taxa of the species. However, in our view, considering its isolated geographical range and its genetic distinction, the population of *L. alpina* growing in the Vranica Mts deserves to be considered as a separate subspecies (according to the subspecies concept given by Stuessy et al. 2014). To our knowledge, no separate taxon has ever been described for this population, probably because of its small and remote geographical range (Fig. 1) and the paucity of relevant herbarium material available (see Table 1). Therefore, the following subspecies is described:

### Leucanthemopsis alpina subsp. vranicae

Taxon classificationPlantaeAsteralesAsteraceae

Tomasello & Konowalik subsp. nov.

B74B4568-D4D2-5C3F-B19D-446C390B7445

urn:lsid:ipni.org:names:77211597-1

#### Description.

Perennial plant, subscapose hemicryptophyte ~ 8–10 cm. Basal leaves pinnatilobate 20–30 mm long and 4–9 mm wide, spatulate with ovate lamina, moderately pilose abaxially, leaflets 5 (–7), 1.5 times longer than width of leaf rachis. Cauline bracts 3 or 4, linear, 3–5 mm long. Inflorescences scapiform, capitula solitary. Involucral bracts green with black margins, pilose over midrib, margin ciliate. Corolla of ray florets white throughout anthesis. Diploid.

#### Diagnosis.

Leucanthemopsis alpina subsp. vranicae differs from subsp. cuneifolia in possessing abaxially pilose leaves (Fig. 3C). In L. alpina subsp. cuneifolia, leaves are glabrous on both surfaces (a few diploid populations from the area surrounding the Dolomites possess abaxially pilose leaves, but are usually smaller plants [up to ca. 5 cm] than those in the Vranica Mts). In L. alpina subsp. vranicae, the green midribs of the involucral bracts are pilose (Fig. 3B), but are glabrous in subsp. cuneifolia. Leucanthemopsis alpina subsp. tatrae leaves are less incised (leaflets up to 1.5 times longer than the width of the leaf rachis), which are tomentose on both surfaces (according to Vierhapper 1914).

#### Distribution and habitat.

Krstac Mt and between Devetaci Mt and Nadkrstac Mt. (Vranica Mts). Bosnia and Herzegovina. Grows solitary or forms clumps on siliceous substrates (gravel and stones) in xerophytic alpine meadows above *Pinus mugo* shrubs. 2000–2100 m a.s.l.

#### Type.

Bosnia and Herzegovina, Central Bosnia Canton, between municipalities of Gornji Vakuf-Uskoplje and Fojnica, close to the village Prokos at the Prokoško jezero lake, Vranica Planina Mountains, Krstac Mt; 43.954833N, 17.736917E; alt. 2036 m; 7 Aug 2018; *K. Konowalik 20180807-02*; (holotype: WRSL [WR GN 064834]; paratypes: WU [WU0043817], SARA [SARA43310, SARA43311, SARA43312]).

#### Conservation status.

Leucanthemopsis alpina subsp. vranicae is a point endemic with a very small range (less than 2.5 km^2^) which meets the IUCN Red List criteria for critically endangered species (CR B2a, IUCN Standards and Petition Committe 2019). Though its population size is not known, it is expected to be low (i.e. during the survey in 2018, only ca. 25 flowering plants were seen in both accessible stands). Therefore, it would also meet criterion D (very small and restricted population, IUCN Standards and Petition Committe 2019). As the subspecies is confined to an alpine habitat, which is heavily restricted in the Vranica Mts, it may face extinction in case of any stochastic event or effects of anthropogenic climate warming. Though, a long time span of collections (130 years) and certain features of those mountains (e.g. microhabitats, inaccessible slopes) may indicate that it will manage to persist there.

##### Identification key to subspecies of *Leucanthemopsis alpina* (modified from Tomasello and Oberprieler 2017).

**Table:** 

1	Dwarf, caespitose and subscapose perennial; leaves palmatifid, villose-tomentose, ca. 10 mm long; leaf segments not spaced; scape short, with 0 (or 1) linear cauline bract; corolla of ray florets reddish during anthesis	**subsp. tomentosa (Loisel.) Heywood**
–	Plants without a dwarf habit; leaves pinnatisect or pinnatilobate; scape usually with 1 or more cauline bracts	**2**
2	Leaves pinnatisect, segments > 2× as long as width of leaf rachis	**3**
–	Leaves pinnatilobate, segments as long or < 2× as long as width of leaf	**6**
3	Plants sericeous-tomentose; corolla of ray florets becoming reddish during anthesis	**subsp. pseudotomentosa (Fiori) Tomasello & Oberpr.**
–	Plants glabrous or moderately pilose, never sericeous-tomentose; corolla of ray florets white or reddish	**4**
4	Leaflets narrow, 3.0–4.5× longer than wide, 2.5–4.0× longer than width of leaf rachis; corolla of ray florets sometimes becoming reddish after anthesis	**subsp. pyrenaica (Vierh.) Tomasello & Oberpr.**
–	Leaflets broader, up to 2.5× longer than width of leaf rachis; corolla of ray florets always remaining white	**5**
5	Plants generally minute (5–10 cm), moderately pilose; leaves 15–20(-25) mm long with 5 to 7 segments; scape with 2 to 4 cauline bracts	**subsp. minima (Vill.) Holub**
–	Plants larger (8–15 cm), glabrous; leaves 20–30 mm long, usually with 7 to 9 segments; scape with 1 to 3 cauline bracts	**subsp. alpina (L.) Heywood**
6	Leaves pubescent on both surfaces; leaflets 1.0–1.5× longer than width of leaf rachis	**subsp. tatrae (Vierh.) Holub**
–	Leaves glabrous or pilose abaxially; leaflets 1.5–2.0× longer than width of leaf rachis	**7**
7	Leaves mostly glabrous; leaflets 1.5–2.0× longer than rachis width; green midribs of involucral bracts glabrous	**subsp. cuneifolia (Murr) Tomasello & Oberpr.**
–	Leaves moderately pilose abaxially; leaflets 1.5× longer than width of leaf rachis; green midribs of involucral bracts pilose	**subsp. vranicae Tomasello & Konowalik**

## Supplementary Material

XML Treatment for Leucanthemopsis alpina subsp. vranicae

## Supplementary materials

Supplementary material 1 Table S1. Table including information on the populations used for inferring the haplotype network (Fig. 2) Data type species data Explanation note Information is given on the population code (used in the lab) collection place, collection number, mountain area, country, geographical coordinates, ploidy and subspecific membership. GenBank accessions codes are provided for the two marker regions employed and for all the samples used for the network reconstruction. https://binary.pensoft.net/file/452674This dataset is made available under the Open Database License (http://opendatacommons.org/licenses/odbl/1.0/). The Open Database License (ODbL) is a license agreement intended to allow users to freely share, modify, and use this Dataset while maintaining this same freedom for others, provided that the original source and author(s) are credited.Salvatore Tomasello, Kamil Konowalik
